# Plasma platelet-derived microparticles to platelet count ratio as a marker of mortality in critically ill patients

**DOI:** 10.1186/cc13400

**Published:** 2014-03-17

**Authors:** M Ohuchi, K Hashimoto, A Ushiba, T Kishimoto, T Yamane, T Hamamoto, T Tabata, Y Tsujita, M Matsushiga, K Takahashi, K Matsumura, K Fujino, Y Eguchi

**Affiliations:** 1 Shiga University of Medical Science, Otsu-shi, Japan

## Introduction

While the crosstalk between coagulopathy and inflammation plays a key role in the development of multiple organ dysfunction in critically ill patients, the mechanisms governing this crosstalk have yet to be established. Microparticles (MPs) are submicron vesicles shed from a variety of cells that are considered to have proinflammatory and prothrombotic properties. Although platelet- derived MPs (PDMPs) are the main form of MPs, the role of PDMPs in critically ill patients remains unclear [1]. The aims of this study were to investigate serum PDMP levels in critically ill patients and to assess their prognostic value.

## Methods

This study comprised 119 critically ill patients who were admitted to the ICU. PDMPs were measured by ELISA three times a week, and 372 samples were obtained. We calculated both the mean PDMP value and the mean PDMP/platelet (PDMP/PLT) ratio (converted to plasma PDMP levels per 10^4 ^platelets) during the course of the ICU stay. Baseline patient data, including APACHE II score, SOFA score, Japanese Association for Acute Medicine DIC score, International Society for Thrombosis and Haemostasis overt DIC score, blood coagulation parameters, C-reactive protein and lactate, were collected at the time of admission to the ICU. The primary outcome was inhospital mortality. Potential predictors were analyzed for possible association with outcomes.

## Results

The mean PDMP/PLT ratio was significantly different when comparing hospital survivors (*n *= 98; median, 1.95) and nonsurvivors (*n *= 21; median 8.40; *P *= 0.00). The mean PDMP/PLT ratio was significantly higher in patients with (median, 4.28) than in those without DiC (median, 1.57; *P *= 0.00). In DIC patients, the mean PDMP/ PLT ratio was significantly higher in nonsurvivors (median, 9.39) than in survivors (median, 3.65; *P *= 0.02). Multivariate logistic regression analysis revealed that both the mean PDMP/PLT ratio (*P *= 0.04) and APACHE II score (*P *= 0.00) were independently associated with in-hospital mortality. The AUCs were calculated as 0.768 ± 0.073 for the mean PDMP/PLT ratio (*P *= 0.00) and 0.811 ± 0.048 for the APACHE II score (*P *= 0.00).

## Conclusion

The PDMP/PLT ratio is a good predictor of in-hospital mortality in critically ill patients, especially in patients with DIC.
